# Tailored Combinations of Human Milk Oligosaccharides Modulate the Immune Response in an In Vitro Model of Intestinal Inflammation

**DOI:** 10.3390/biom14121481

**Published:** 2024-11-21

**Authors:** Clodagh Walsh, Jonathan A. Lane, Douwe van Sinderen, Rita M. Hickey

**Affiliations:** 1 Teagasc Food Research Centre, Moorepark, Fermoy, P61 C996 Cork, Ireland; clodagh.walsh@hh.global; 2 Health and Happiness Group, H&H Research, P61 K202 Cork, Ireland; jonathan@hh.global; 3 APC Microbiome Ireland and School of Microbiology, University College Cork, T12 YT20 Cork, Ireland; d.vansinderen@ucc.ie

**Keywords:** HMO, immunity, inflammation, NEC, prebiotics, breastmilk, Caco-2

## Abstract

Infants rely on their developing immune system and the protective components of breast milk to defend against bacterial and viral pathogens, as well as immune disorders such as food allergies, prior to the introduction of solid foods. When breastfeeding is not feasible, fortified infant formula will most frequently be offered, usually based on a cow’s milk-based substitute. The current study aimed to explore the immunomodulatory effects of combinations of commercially available human milk oligosaccharides (HMOs). An in vitro co-culture model of Caco-2 intestinal epithelial cells and THP-1 macrophages was established to replicate the hallmarks of intestinal inflammation and to evaluate the direct effects of different synthetic HMO combinations. Notably, a blend of the most prevalent fucosylated and sialylated HMOs, 2′-fucosyllactose (2′-FL) and 6′-siallylactose (6′-SL), respectively, resulted in decreased pro-inflammatory cytokine levels. These effects were dependent on the HMO concentration and on the HMO ratio resembling those in breastmilk. Interestingly, adding additional HMO structures did not enhance the anti-inflammatory effects. This research highlights the importance of carefully selecting HMO combinations in nutritional products, particularly for infant milk formulations, to effectively mimic the benefits associated with breastmilk.

## 1. Introduction

To protect against foreign invaders, humans have evolved intricate defence mechanisms, collectively known as the immune system. Immune system development begins before birth and continues postnatally as the body encounters new molecular signals. Shortly after birth, a newborn’s immune system undergoes rapid changes, with various immune cells—including neutrophils, macrophages, monocytes, natural killer (NK) cells, T cells, and dendritic cells—actively participating in the defense against pathogenic invaders [1,2]. At birth, there is an observable imbalance in T-helper (Th) cell types, specifically between Th1/17 and Th2 phenotypes. In neonates, the immune response leans towards the Th2 phenotype, which enhances humoral immunity and provides protection against extracellular pathogens [3,4]. Conversely, pathways associated with Th1/Th17, which target intracellular pathogens, are less prominent in early neonatal development [5,6]. Disruption of this system by various factors such as stress, nutrient deficiencies, environmental factors or exposure to pathogens can lead to inflammation, a fundamental response to foreign materials. Inflammatory reactions involve the secretion of chemokines and cytokines attracting immune cells such as macrophages and T-cells, which act against the pathogen [7]. However, dysregulated immune responses can lead to excessive inflammation, damaging healthy tissues and contributing to the emergence of inflammatory diseases, including necrotizing enterocolitis (NEC) [8,9]. Breastfeeding has been shown to significantly decrease the risk of these conditions, implying that human milk has components supporting the maturation of the gut and immune system [10,11].

Human milk oligosaccharides (HMOs), which represent a diverse group of lactose-based sugars, are the third-largest solid component in breastmilk that have been shown to play a crucial role in early life development [12,13]. Although extensively studied for their prebiotic effects, through promoting growth of beneficial bacteria in the gut [14,15], HMOs also offer a broader range of health benefits. These sugars can influence gut motility, exhibit anti-adhesive and antimicrobial properties, and regulate epithelial cell responses [16,17,18]. The advantages of HMOs may extend beyond infancy, potentially reducing allergy risk or supporting cognitive function [19,20,21], which has made HMOs the focus of intense current scientific research with an increasing number of studies unravelling their role in human physiology. Over the past decade, substantial clinical evidence has highlighted HMO benefits across various populations (as evaluated in a recent systemic review by Schönknecht et al. [22]) across varying study populations including healthy infants [23,24,25,26], infants with specific health needs [27,28,29], children [30], and adults [31]. While HMOs are believed to support immune system development by aiding the establishment of a healthy gut microbiome and its metabolic functions, recent studies also suggest that HMOs can directly impact immune responses [32].

HMOs have been found to exhibit anti-inflammatory activity by regulating the production of interleukins (ILs) and lymphocyte activation [33]. He et al. utilized intestinal epithelial cells (T84, HCT8, FHs74) and HeLa cells to explore the immunomodulatory effects of human milk oligosaccharides (HMOs) derived from colostrum [34]. Their research identified key networks involved in immune cell communication, differentiation of the intestinal mucosal immune system, and maintenance of homeostasis. HMO treatment resulted in a reduction in pro-inflammatory cytokine levels, including IL-8, IL-6, monocyte chemoattractant protein-1/2, and IL-1β. Conversely, it elevated the levels of cytokines associated with tissue repair and homeostasis, highlighting the potential of HMOs to modulate immune responses and promote intestinal health [34]. In a similar study, the transcriptional response of colonic epithelial cells treated with human milk oligosaccharides (HMOs) was examined using human colorectal adenocarcinoma HT-29 cells. The treatment with breastmilk-derived HMOs was shown to significantly affect expression of several cytokines, including IL-1β, IL-8, IL-17C, and platelet factor 4 (PF4). Additionally, the levels of various chemokines, such as CXCL1, CXCL2, CXCL3, CXCL6, CCL5, CCL20, and CX3CL1, as well as the cell surface receptor interferon γ receptor 1 (IFNGR1), were also modulated by HMO administration [35]. Newburg et al. revealed that HMO isolated from breastmilk attenuated TNF-α and IL-1β-induced expression of IL-8, monocyte chemoattractant protein 1 (MCP-1), and macrophage inflammatory protein-3α (MIP-3α) [36]. These findings underscore the role of breastmilk-derived pools of HMOs in regulating immune signalling pathways in colonic epithelial cells. However, these anti-inflammatory effects have also been demonstrated for individual HMO structures. Recent advancements in synthetic biology and metabolic engineering have enabled microbial systems to produce various HMOs, facilitating their use in both commercial and research settings. He et al. examined the influence of 2′-fucosyllactose (2′-FL), one of the most prominent short-chain oligosaccharides in milk, on *Escherichia coli*-induced interleukin (IL)-8 release by T84 and H4 intestinal epithelial cells [34]. The study demonstrated that 2′-FL quenches pro-inflammatory signalling through attenuation of CD14 induction in this in vitro model [34]. These HMO-induced anti-inflammatory effects were also observed in human epithelial cell responses related to allergic disease [37]. For example, Zehra et al. investigated the therapeutic potential of specific HMOs in food allergy and demonstrated that the HMO, 6′-sialyllactose (6′-SL), inhibited chemokine (IL-8 and CCL20) release from lung T-84 and intestinal HT-29 cells stimulated with antigen–antibody complex [37]. This effect was PPARγ-dependent, while 2′-FL selectively inhibited CCL20 release in response to antigen–antibody complex in a PPARγ-independent manner [37]. In addition, sialylated HMOs suppressed inflammatory cytokine (IL-1β, TNF-α and IL-6) production and reduced NEC incidence and pathological damages in inflamed ileum of NEC rats in vivo [38]. It is clear, therefore, that HMOs have structure-specific impacts, with clear distinctions between neutral and acidic oligosaccharides, in modulating cytokine production and cell differentiation.

While previous in vitro studies indicate the potential of HMOs as anti-inflammatory agents in intestinal settings, research has largely focused on individual HMO structures. Blends of synthetic HMOs can mimic the diverse mixture found in natural breastmilk more closely, potentially offering a broader range of benefits. Synergy between specific HMO combinations may enhance immune modulation, with combined effects on reducing inflammation, improving gut barrier integrity, and supporting overall immune development. This study aims to explore whether specific combinations of commercial HMO ingredients can replicate the immunomodulatory effects of natural HMO pools. A co-culture model of Caco-2 intestinal epithelial cells and THP-1 macrophages was developed in order to mimic the hallmarks of intestinal inflammation and used to investigate the effects of different synthetic HMO combinations. Concentration and ratio dependency assays were performed to elucidate the functional synergy between HMO structures in mitigating inflammation.

## 2. Materials and Methods

### 2.1. Materials

Cell culture mediums (DMEM, RPMI-1640, PBS), fetal bovine serum (FBS), trypsin/EDTA, antibiotics (penicillin/streptomycin solution), human interferon-γ (IFN-γ), MILLIPLEX^®^ Human High Sensitivity T Cell Panel, and the Lactate Dehydrogenase Activity Assay Kit were supplied by Sigma-Aldrich (St Louis, MO, USA). Lipopolysaccharide (LPS) derived from *Escherichia coli* O55:B5 and obtained from Sigma-Aldrich. Phenol red-free DMEM was obtained from GIBCO (Carlsbad, CA, USA). CellTiter 96^®^ Aqueous One Solution Reagent was purchased from Promega (Madison, WI, USA). Phorbol 12-Myristate 13-Acetate (PMA) was obtained from VWR (Radnor, PA, USA).

### 2.2. Ingredient Generation and Selection

#### 2.2.1. Selection of Commercial HMO Ingredients

Commercial HMO powders were received from Glycom A/S (Hørsholm, Denmark, purity > 90%). For use in experiments, HMO ingredients had to fulfill the following criteria: (a) a positive response as a novel food ingredient from European Food Safety Authority (EFSA) as of 2022; (b) endotoxin levels < 0.025 EU/mg; (c) no cytotoxic effects at concentrations specified in Table 1. HMO or control ingredients were dissolved in glucose-free, phenol red-free media (Fisher, Waltham, MA, USA) and applied throughout experiments at a concentration relative to the regulatory concentration outlined in EFSA’s Union list of novel foods (https://food.ec.europa.eu/food-safety/novel-food/authorisations/union-list-novel-foods_en accessed on 30 July 2024).

#### 2.2.2. Generation of HMO-Enriched Fraction

Human milk samples of unknown secretor status were generously provided by the Irvinestown Human Milk Bank (Co. Fermanagh, Ireland) and were stored at −80 °C upon arrival. Briefly, lipids and proteins were removed from breastmilk as previously described [39], after which lactose and HMO fractions were separated using size-exclusion chromatography (BioGel P2, 92 cm × 5 cm; Bio-Rad Laboratories, Inc., Hercules, CA, USA). Fractions confirmed to be free of peptides (assessed with the Pierce colorimetric peptide assay) and containing minimal lactose (measured using high-pH anion exchange chromatography [HPAEC] with pulsed amperometric detection [Dionex Corporation, Sunnyvale, CA, USA]) were combined and freeze-dried. The resulting HMO-rich fraction was stored at 4 °C until use in experiments. Analysis by HPAEC revealed small amounts of 2′-fucosyllactose (2′-FL) in the HMO-enriched fraction, likely due to the co-fractionation of 2′-FL with lactose during purification. To restore the 2′-FL content that may have been lost in the size-exclusion process, commercial 2′-FL was reintroduced, following a previously described method [40], to give a powder representative of secretor HMO (designated as breastmilk-derived HMO). The composition of the breastmilk-derived HMO is shown in Supplementary Figure S1.

### 2.3. Cell Culture

#### 2.3.1. Cell Line Maintenance

An in vitro model using co-cultures of Caco-2 and PMA-differentiated THP-1 cells was established and optimized to mimic the intestine in an inflammatory state based on methods previously outlined [41]. Caco-2 cells were obtained from the American Type Culture Collection (ATCC) and cultured in DMEM-based cell culture medium (phenol red-free, Merck, Darmstadt, Germany) substituted with 10% heat-inactivated FBS (Merck) and 1% Penicillin/Streptomycin at 37 °C, 5% CO_2_. For co-cultures, Caco-2 cells were seeded on transwell inserts (0.4 μm pore size, Corning) at a density of 6 × 10^4^ cells/well and maintained for 21 days. On the apical (AP) side, the cells were cultured in DMEM, whereas the medium in the basolateral (BL) compartment was changed to RPMI-based medium (10% FBS). Caco-2 cells were used for experiments at three different passages (passage numbers 41 to 45) to provide three biological replicates. THP-1 cells were purchased from the European Collection of Authenticated Cell Cultures (ECACC) and cultured at 37 °C, 5% CO_2_ using RPMI-1640 medium substituted with 10% heat-inactivated FBS and 0.1% mercaptoethanol. For co-culture experiments, THP-1 cells were seeded on transwell-suitable 12-well plates at a density of cells/well and differentiated with PMA (100 nM) for 24 h (37 °C, 5% CO_2_). Following differentiation, the PMA was removed, and the THP-1 cells cultured for a further 24 h before use in experiments. THP-1 cells were used for experiments at three different passages (passage numbers 4 to 6) to provide three biological replicates.

#### 2.3.2. Cytotoxic/Proliferative Effects of HMO Ingredients

To assess the cytotoxicity of HMO ingredients, the CellTiter 96 Aqueous One Solution Cell Proliferation Assay (Promega, Madison, WI, USA) was employed. This colorimetric method quantifies the number of viable cells. Caco-2 cells were seeded at a concentration of 5 × 10^4^ cells per well in a 96-well plate and incubated for 24 h with DMEM (10% FBS). Following cell attachment, the spent media were aspirated, and cells were rinsed twice with PBS. Subsequently, cells were treated with 500 µL HMO solutions for 24 h. HMO solutions were prepared by dissolving ingredients in DMEM (without glucose or phenol red, GIBCO) at low (0.5× regulatory concentration), standard (1× regulatory concentration), and high (2× regulatory concentration) doses. A control group with DMEM medium without HMOs was included. After the 24 h treatment, cells were exposed to 20 µL/well of CellTiter 96 Aqueous One Solution Reagent, which contains MTS tetrazolium. The MTS tetrazolium compound is enzymatically converted by viable cells into a colored formazan product. Following a 2 h incubation at 37 °C, absorbance was measured at 490 nm using a 96-well plate reader. Each experiment was conducted in duplicate, and the recorded values of the HMO solutions were adjusted based on the results obtained from the control well.

### 2.4. Cell Culture

#### 2.4.1. Induction of Inflammation-like Response

**Figure 1:**
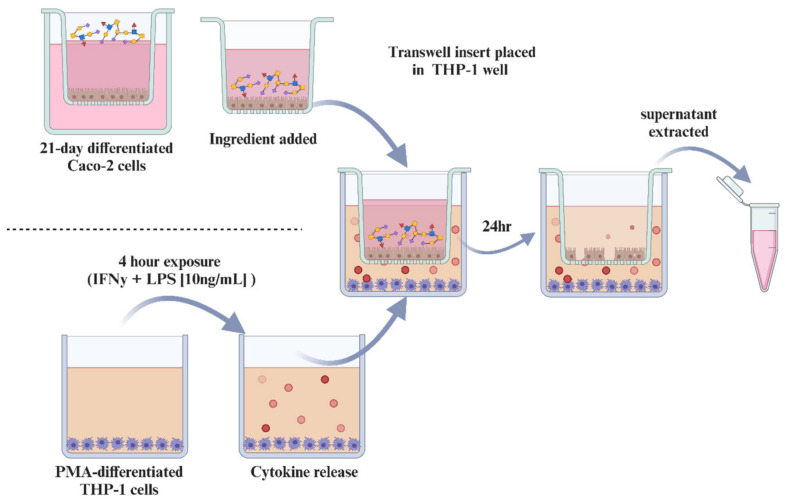
Schematic description of the in vitro model using co-cultures of Caco-2 and PMA-differentiated THP-1 cells to mimic the intestine in an inflammatory state.

An in vitro model using co-cultures of Caco-2 and PMA-differentiated THP-1 cells was established (see Figure 1) and optimized to mimic the intestine in an inflammatory state based on previously described methods [41]. THP-1 cells were pre-stimulated with LPS and IFN-γ (1, 10, or 100 ng/mL each) for 4 h. IEC Caco-2 transwells were then transferred into THP-1 wells and incubated with primed THP-1 cells for 24 h at 37 °C, 5% CO_2_. Control wells containing (a) Caco-2 and THP-1 cells without stimulation and (b) Caco-2 cells in monoculture were included in experiments. Following this 24 h incubation period, supernatants from the Caco-2 apical compartment were collected in polypropylene tubes and stored at −20 °C for quantification of inflammatory cytokines (as outlined below). In addition, lactate dehydrogenase (LDH) activity in the cell culture medium was measured after 24 h co-culture using a Lactate Dehydrogenase Activity Assay Kit (Sigma-Aldrich) according to manufacturer’s instructions. The TEER was measured using an Ohm-meter (EVOM2) to assess the barrier development of the Caco-2 cell layer (every 6–7 days of culture), as well as the barrier integrity throughout the co-culture with THP-1 cells (after 4, 18, 24, and 48 h). The electrode was sterilized in 70% ethanol (15 min) and neutralized in PBS. Results were corrected for the blank and multiplied by the membrane filter area (1.12 cm^2^) to obtain the final results in Ohm per cm^2^ (Ω·cm^2^).

The following criteria for the co-culture to be classified as ‘inflamed’ were defined:

1. A TEER reduction by at least 25% compared to the Caco-2 monoculture.
2. After 24 h of co-culture, the levels of pro-inflammatory cytokines need to significantly exceed the concentrations recovered in the stable co-culture (Caco-2 and THP-1 cells without stimulation).
3. Re-establishment of TEER after 48 h of co-culture (>90% recovery to TEER values at initiation of co-culture).
4. Significantly lower levels of LDH activity (as measured by absorbance at 490 nm).

#### 2.4.2. Exposure of Caco-2 IECs to HMO Ingredients

Following selection of optimal stimulation (10 ng/mL LPS and IFN-γ), HMO ingredients were introduced to the system and changes in cytokine expression assessed. HMO ingredients were prepared as outlined below (to assess structure dependency, concentration dependency, and ratio dependency effects) and added to the Caco-2 monolayers, before transferring the Caco-2 transwells into the pre-stimulated THP-1 wells. Caco-2 cells were incubated with primed THP-1 cells and ingredients for 24 h at 37 °C, 5% CO_2_. Following this 24 h incubation period, supernatants from the Caco-2 apical compartment were collected in polypropylene tubes and stored at −20 °C.

##### Structure Dependency Assay

Initially, the anti-inflammatory effects of HMO combinations were assessed and compared to the anti-inflammatory properties of individual HMOs. HMO ingredients were dissolved in DMEM media at maximum regulatory concentrations as outlined in Table 1. Media supplemented with glucose or breastmilk-derived HMO (at equivalent concentrations to synthetic HMO) were used as controls. After 24 h co-exposure of the Caco-2 transwells to the HMO ingredients and activated THP-1, supernatants were collected and the release of IL-8 in the apical compartment was quantified using IL-8 ELISA (Biolegend). The absorbance was read spectrophotometrically at 450 nm.

##### Concentration Dependency Assay

To further understand the immunomodulatory effects of these HMO ingredients, the co-culture experiments were repeated using various concentrations of 2′-FL (0.6–2.4 g/L) and 6′-SL (0.2–0.8 g/L). These were applied as singular doses, in combination with each other (2′-FL + 6′-SL), and in combination with LNFP-I, LNT, and LNnT (5-HMO) (Table 2). All HMOs were applied at low, standard, and high doses equivalent to 0.5×, 1×, and 2× the maximum regulated dose (according to the European Union List of authorized novel foods). The Caco-2 transwells were co-exposed to the activated THP-1 cells and the HMO ingredients for 24 h before collecting the supernatants from the apical compartment and measuring cytokine levels. The release of TNF-α, IL-8, IL-10, IL-6, and IL-1β was quantified using a magnetic bead-based assay (Milliplex HSTCMAG-28SK high-sensitivity panel, Merck Millipore, Burlington, MA, USA) and analyzed with the Bio-Plex MAGPIX Multiplex Reader (Luminex). For the analysis, supernatants from the inflamed model were diluted 1:20 in cell culture media. Samples were added to appropriate wells and incubated overnight at 4 °C. Subsequently, the detection antibodies were added, and the plate incubated again for 1 h at RT. The wells were incubated with streptavidin-PE and incubated for 30 min at RT before the beads were re-suspended in assay buffer and the plate read using a Bio-Plex MAGPIX Multiplex Reader. Blanks and standard curves were included on each plate.

##### Ratio Dependency Assay

To assess whether the potency of HMO could be modulated by adjusting the ratio of HMO components, the immune model was treated with various combinations of 2′-FL and 6′-SL provided in various ratios ranging from 1:2 to 3 to 6:1 according to Table 3. The equivalent concentrations of individual 2′-FL and 6′-SL were included for comparative purposes. The release of TNF-α, IL-8, IL-10, IL-6, and IL-1β were quantified using a magnetic bead-based assay (Milliplex HSTCMAG-28SK high-sensitivity panel). In addition, the effects of these combinations on barrier integrity were assessed using TEER as an indicative measurement. The TEER was measured using an Ohm-meter. Results were corrected for the blank and multiplied by the membrane filter area (1.12 cm^2^) to obtain the final results in Ohm per cm^2^ (Ω·cm^2^).

### 2.5. Cytokine Quantification

#### 2.5.1. Enzyme-Linked Immuno-Sorbent Assay (ELISA)

The release of IL-8 in the apical compartment was quantified after 24 h co-culture following pre- and co-exposure to ingredients. The ELISA was run according to manufacturer’s instructions (Biolegend, San Diego, CA, USA). The absorbance was read spectrophotometrically at 450 nm.

#### 2.5.2. Bio-Plex Magpix

Release of TNF-α, IL-8, IL-10, IL-6, or IL-1β was quantified using a magnetic bead-based assay (Milliplex HSTCMAG-28SK high-sensitivity panel, Merck Millipore) and analyzed with the Bio-Plex MAGPIX Multiplex Reader (Luminex, Austin, TX, USA). For the analysis, supernatants from the inflamed model were diluted 1:20 in cell culture media. Samples were added to appropriate wells and incubated overnight at 4 °C. Subsequently, the detection antibodies were added and the plate incubated again for 1 h at RT. The wells were incubated with streptavidin-PE and incubated for 30 min at RT before the beads were re-suspended in assay buffer and the plate read using a Bio-Plex MAGPIX Multiplex Reader. Blanks and standard curves were included on each plate.

### 2.6. Statistical Analysis

Experimental results are expressed as means ± the standard errors of the means. Statistical analyses were performed by means of one-way analysis of variance (ANOVA), followed by Tukey’s test for comparing all pairs of groups. Significant differences between 2 groups were determined using the unpaired Student’s *t*-test. A statistical software package (GraphPad PRISM™ version 5.0, GraphPad Software Inc, La Jolla, CA, USA) was used for the analysis. A *p*-value of <0.05 was considered statistically significant.

## 3. Results

### 3.1. Cytotoxic and Proliferative Effects of HMO Ingredients

**Figure 2:**
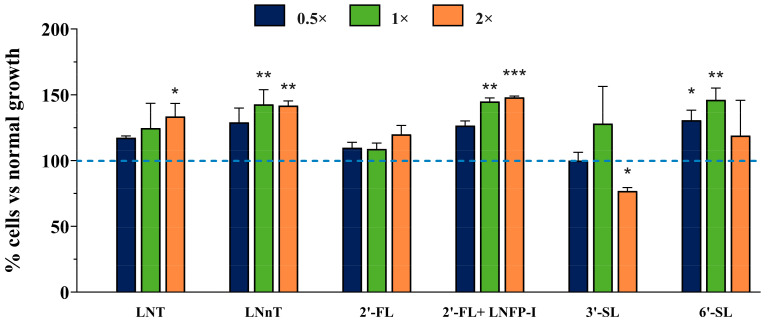
Impact of HMO treatments on Caco-2 cell proliferation and viability assessed using the CellTiter 96 Aqueous One Solution Cell Proliferation Assay. HMOs were applied at low (0.5× regulatory concentration, navy), standard (1× regulatory concentration, green), and high (2× regulatory concentration, orange) doses. Proliferation rates were compared to the control group (cell culture medium without HMO, set at 100%, indicated by the dotted blue line). LNT = Lacto-*N*-tetraose, LNnT = Lacto-*N*-neotetraose, 2′-FL = 2′-fucosyllactose, LNFP-I = Lacto-*N*-fucopentaose-I, 3′-SL = 3′-sialyllactose, 6′-SL = 6′-sialyllactose. Univariate analysis of variance (ANOVA) and post hoc Tukey tests were performed to determine the significant differences between treatment groups and the control group (* *p* < 0.05, ** *p* < 0.01, *** *p* < 0.001, vs. control). Analysis was performed using technical duplicate data from biological triplicate experiments (passage numbers 41–45), and the data are presented as means ± SD.

The effect of each treatment of various HMO combinations/concentrations (2′-FL, LNFP-I, LNT, LNnT, 3′-SL, and/or 6′-SL at various concentrations) on Caco-2 proliferation is summarized in Figure 2. All comparisons of proliferation rates were made relative to the control group (cell culture medium without HMO exposure, designated with a value of 100%). The results showed that the majority of HMO treatments did not affect the viability of Caco-2 cells (Figure 1). In fact, increased proliferation of Caco-2 cells was observed in the presence of LNT at 2× (*p* = 0.0185), LNnT at 1× (*p* = 0.0023), LNnT at 2× (*p* = 0.0028), LNFP-I at 1× (*p* = 0.0013), LNFP-I at 2× (*p* = 0.0006), 6′-SL at 0.5× (*p* = 0.0342), and 6′-SL at 1× (*p* = 0.0010). In contrast, when Caco-2 cells were incubated with 0.4 g/L (2×) of 3′-SL (Figure 2), the number of viable cells decreased to 76.9 ± 2.55%, indicating potential cytotoxic effects of 3′-SL at higher concentrations. This HMO was thereby eliminated from further analyses.

### 3.2. Optimization of Inflamed Co-Culture

For the co-culture setup, transwell filters with Caco-2 cells were placed into well plates containing THP-1 cells that had been differentiated over 24 h and stimulated with varying concentrations of LPS and IFNγ (0 ng/mL for stable co-culture, 1 ng/mL, 10 ng/mL, and 100 ng/mL). To determine the most effective concentration of these immune stimulants, the functional characteristics, cellular integrity, and cytokine release in the inflamed co-culture models were compared with those in a Caco-2 monoculture.

**Figure 3:**
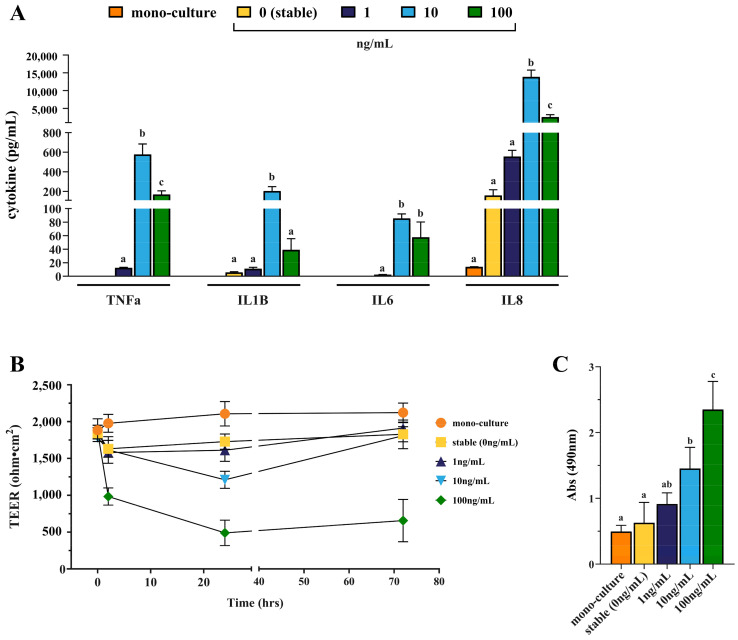
(**A**) Release of pro-inflammatory cytokines (TNFα, IL-1β, IL-6, and IL-8) after 24 h Caco-2 monoculture (orange), stable THP-1 co-culture (no LPS, yellow) or inflamed THP-1 co-culture following treatment with 1 (navy), 10 (blue), and 100 (green) ng/mL of both LPS and IFNγ, as measured by Milliplex HSTCMAG-28SK high-sensitivity panel. Values for each cytokine with common letter = *p* > 0.05, calculated using Tukey’s multiple comparisons test. (**B**) Change in transepithelial electrical resistance after 24 h inflammation followed by 48 h recovery period (total 72 h) as measured using an EVOM2 Ohm-meter. Measurements were adjusted for the blank and calculated per membrane area (1.12 cm^2^), yielding results in Ohm per cm^2^ (Ω·cm^2^). (**C**) LDH release following 24 h in Caco-2 monoculture, stable, and inflamed co-cultures, detected using an LDH assay kit and measured spectrophotometrically at 490 nm. (**C**) LDH release after 24 h Caco-2 monoculture, stable, and inflamed co-culture, using LDH detection kit and measuring spectrophotometrically at 490 nm. Values with common letter = *p* > 0.05, calculated using Tukey’s multiple comparisons test. Analysis was performed using technical triplicate data from biological triplicate experiments (passage numbers 41–45 for Caco-2 cells, passage numbers 4–6 for THP-1 cells), and the data are presented as means ± SD.

Initially, cytokine levels in the apical compartment from the Caco-2 monoculture and co-culture models were assessed using a MAGPIX magnetic bead-based assay. As summarized in Figure 3A, the levels of TNF-α, IL-1β, IL-6, and IL-8 released after 24 h culturing varied significantly depending on the presence or absence of THP-1 cells and on the levels of immunostimulant present. In general, baseline cytokine levels in the Caco-2 monoculture were below the limit of detection, although IL-8 was expressed at low concentrations (13.9 ± 0.11 pg/mL). These levels of IL-8 increased to 159.8 ± 57.68 pg/mL when the Caco-2 cells were co-cultured with THP-1 cells under stable conditions, with low levels of IL-1β also detected (5.7 ± 0.99 pg/mL). The inflamed co-culture models showed marked cytokine release compared to both the monoculture and stable co-culture, although responses to 1 ng/mL LPS + IFN-γ were relatively modest (12.5 pg/mL TNF-α, 11.0 pg/mL IL-1B, 2.3 pg/mL IL-6, and 556.5 pg/mL IL-8). In contrast, stimulation of THP-1 cells with 10 ng/mL LPS + IFN-γ resulted in the release of 577.7 pg/mL of TNF-α, 205.3 pg/mL of IL-1β, and 85.5 pg/mL of IL-6. Most strikingly, the levels of IL-8 detected in the Caco-2 supernatants increased to 13,843.3 pg/mL in response to 10 ng/mL LPS + IFN-γ stimulation (Figure 3A). These concentrations of pro-inflammatory cytokines were therefore sufficient to meet the criteria defined for an inflamed model. Surprisingly, however, increasing the concentration of LPS + IFN-γ to 100 ng/mL did not result in significant increases in cytokine concentrations. As shown in Figure 3A, high doses of LPS + IFN-γ resulted in significantly lower levels of TNF-α, IL-1β, and IL-8 (*p* < 0.01) when compared to stimulation with 10 ng/mL LPS + IFN-γ.

The barrier integrity of the Caco-2 cells was assessed by measuring TEER at 0, 2, and 24 h of co-culture. In addition, the ability of the Caco-2 IECs to re-establish was assessed by measuring TEER 48 h after the co-culture experiments (corresponding to 72 h after initiation of experiments). As summarized in Figure 3B, the stable co-culture of Caco-2 cells and THP-1 cells induced a slight reduction in TEER over a 24 h period. When compared to Caco-2 in monoculture, the TEER reduced from 2106.1 ± 166.16 Ω·cm^2^ to 1728.1 ± 102.76 Ω·cm^2^ in the stable co-culture (corresponding to 82.1% TEER of the monoculture control, *p* = 0.0141). Activation of inflammation in the THP-1 cells using 1 ng/mL LPS + IFN-γ did not induce a significant reduction in TEER when compared to the stable culture (1611.5 ± 151.18 Ω·cm^2^, *p* = 0.837), whereas stimulation with 10 ng/mL and 100 ng/mL LPS + IFN-γ resulted in significant barrier disruption (Figure 3B). After 24 h, the TEER in the inflamed co-cultures using 10 ng/mL and 100 ng/mL LPS + IFN-γ was 57.4% (*p* < 0.0001) and 23.2% (*p* < 0.0001), respectively, versus the monoculture control (Figure 3B). The barrier disruption was therefore sufficient to meet the criteria defined for an inflamed model (>25%). Notably, however, the barrier integrity of the Caco-2 cells in the presence of THP-1 cells activated by 100 ng/mL LPS + IFN-γ did not re-establish to the levels of the Caco-2 monoculture after 48 h (655.8± 287.80 Ω·cm^2^, *p* < 0.0001), suggesting that excessive cell death had occurred. In contrast, the TEER in all other co-cultures (stable, 1 ng/mL, and 10 ng/mL) had re-established to >85% (*p*> 0.05) of the Caco-2 monoculture after 48 h (Figure 3B).

To further investigate cell damage, lactate dehydrogenase (LDH) release was measured, as high levels of THP-1 activation may induce cell death. LDH, a stable cytoplasmic enzyme released when cell membranes are damaged, was quantified by measuring the conversion of NAD to NADH, which absorbs at 490 nm. Low LDH activity (0.5 ± 0.09 A450) was observed in the Caco-2 monoculture (Figure 3C). No significant increase in LDH was observed in the stable co-culture or the 1 ng/mL inflamed co-culture. Activation of inflammation in the THP-1 cells using 10 ng/mL LPS + IFN-γ resulted in large increases (192.6%) in LDH activity. This activity was further increased in the presence of 100 ng/mL LPS + IFN-γ-stimulated THP-1 cells. Such activation resulted in a 373.2% increase in LDH activity, indicating the occurrence of necrotic cell death (Figure 3C). These LDH readings confirm that excessive cell death occurred in Caco-2 intestinal cells during high activation of THP-1 macrophages. In contrast, low doses of LPS + IFN-γ (1 ng/mL) did not induce a sufficiently strong reaction to meet the criteria defined for an inflamed intestine. A concentration of 10 ng/mL LPS + IFN-γ was therefore selected as the optimal concentration to activate THP-1 cells and all subsequent experiments were carried out using this dose.

### 3.3. Influence of Human Milk Oligosaccharides on Cytokine/Chemokine Release

#### 3.3.1. Identifying the Optimal Combination of HMO for Amelioration of Inflammation

**Figure 4:**
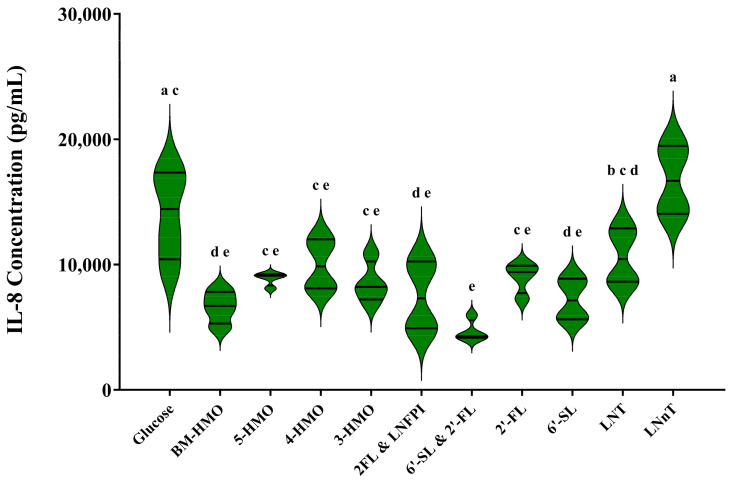
Release of pro-inflammatory IL-8 after 24 h Caco-2 THP-1 inflamed co-culture (induced using 10 ng/mL of both LPS and IFNγ) following treatment with HMO. Concentrations of HMO used in treatments can be found in Table 1. BM-HMO = breastmilk-derived HMO, 2′-FL = 2′-fucosyllactose, LNFP-I = Lacto-*N*-fucopentaose-I, 6′-SL = 6′-sialyllactose, LNT = Lacto-*N*-tetraose, LNnT = Lacto-*N*-neotetraose. Values with common letter = *p* > 0.05, calculated using Tukey’s multiple comparisons test. Analysis was performed using technical triplicate data from biological triplicate experiments (passage numbers 41–45 for Caco-2 cells, passage numbers 4–6 for THP-1 cells), and the data are presented as violin plot showing data distribution, median, and interquartile range.

Following optimization and establishment of the inflamed model (employing 10 ng/mL LPS + IFN-γ), the anti-inflammatory effects of various HMO mixes were assessed and compared to the anti-inflammatory properties of the same HMO applied individually. Breastmilk-derived HMO (BM-HMO) were used as a positive control. As a negative control, DMEM supplemented with glucose at an equivalent concentration to HMO was used. HMOs were applied at the maximum approved concentration as per the European Union List of authorized novel foods, as outlined in Table 1. The quantification of IL-8 using Biolegend ELISA is summarized in Figure 4 and shows that the levels of IL-8 released into Caco-2 supernatants varied significantly depending on the number and specific structure of the HMO(s) applied. Levels of IL-8 expression in the glucose control were 14,059.8 ± 3615.99 pg/mL, while in the BM-HMO-treated cells, levels were 6432.3 ± 555.68 pg/mL (*p* = 0.0036). The majority of the HMO treatments assessed demonstrated a capacity to modulate cytokine expression in the inflamed model. Following disruption of immune homeostasis, it was found that all HMO treatments, aside from LNnT (at 0.6 g/L) (*p* < 0.0001), resulted in similar IL-8 levels to HMO isolated from breastmilk (BM-HMO at 3.8 g/L). Notably, the lowest concentrations of IL-8 were detected in Caco-2 supernatants where a 2-HMO combination of the most prevalent fucosylated (2′-FL at 1.2 g/L) and sialylated (6′-SL at 0.4 g/L) HMO had been applied to the Caco-2 cells. Treatment with this HMO combination reduced the levels of pro-inflammatory IL-8 by 63.7 ± 17.21% when compared to the levels detected in the glucose control (*p* = 0.0001). Similar IL-8 levels were detected following application of a 2-HMO combination of 2′-FL and LNFP-1 (7488.5 pg/mL) as well as a treatment with a 3-HMO blend (8556.5 pg/mL). An increase in the number of HMO structures (4-HMO or 5-HMO) did not significantly reduce IL-8 levels when compared to those detected in the glucose control.

#### 3.3.2. Identifying the Optimal Combination of HMO for Amelioration of Inflammation

**Figure 5:**
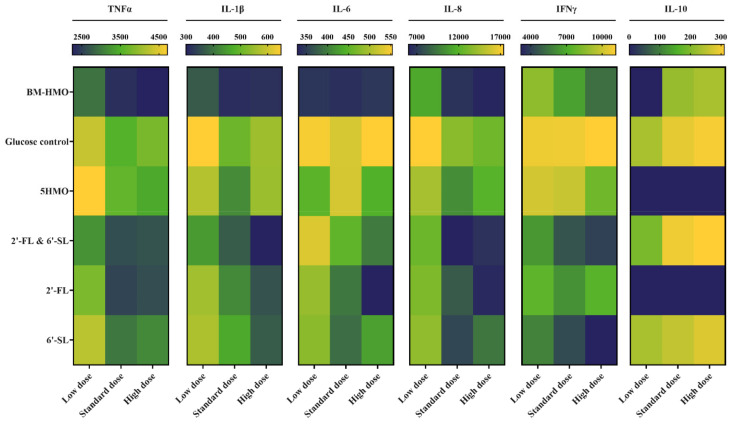
Heatmap summarizing release of pro- and anti-inflammatory cytokines (TNFα, IL-1β, IL-6, IL-8, IFNγ, and IL-10) following 24 h HMO treatment of inflamed Caco-2 THP-1 co-culture (induced using 10 ng/mL of both LPS and IFNγ). Cytokine release was measured using Milliplex HSTCMAG-28SK high-sensitivity panel. Lower concentrations of cytokines are shown in navy, while higher levels of cytokine release are depicted in yellow as per the scale indicated on top of graph. HMOs were applied at low (0.5× regulatory concentration, navy), standard (1× regulatory concentration, green), and high (2× regulatory concentration, orange) doses as per Table 3. BM-HMO = breastmilk-derived HMO, 2′-FL = 2′-fucosyllactose, 6′-SL = 6′-sialyllactose. Analysis was performed using technical triplicate data from biological triplicate experiments (passage numbers 41–45 for Caco-2 cells, passage numbers 4–6 for THP-1 cells).

To determine whether the immunomodulatory effects of these HMO ingredients were concentration-dependent, the co-culture experiments were repeated using various concentrations of 2′-FL (0.6–2.4 g/L) and 6′-SL (0.2–0.8 g/L). These were applied individually, in combination with each other (2′-FL + 6′-SL), and in combination with LNFP-I, LNT, and LNnT (5-HMO) (Table 2). The Caco-2 transwells were co-exposed to the activated THP-1 cells and the HMO ingredients for 24 h before collecting the supernatants from the apical compartment and measuring cytokine levels (IL-1β, IL-6, IL-8, TNF-α, IFNγ, and IL-10) using Magpix ELISA. Anti-inflammatory IL-10 was only detected in Caco-2 supernatants where the inflamed intestinal cells had been exposed to standard and high doses of BM-HMO, glucose, 2′-FL + 6′-SL, or 6′-SL (Figure 5). Pro-inflammatory IL-1β, IL-6, IL-8, TNF-α, and IFNγ were detected in all Caco-2 supernatants (Figure 5). Concentration dependency assays indicated that, in general, the protective effects of HMO treatments lessened at lower doses, but no added benefits were observed when higher doses were applied. This was noted in particular for expression of pro-inflammatory IL-8. Across all treatment groups, higher levels of IL-8 were detected following exposure of Caco-2 cells to low doses (0.5× regulatory doses) of HMO when compared to standard and high doses (1× and 2× regulatory doses, respectively) (*p* < 0.05). No significant difference in IL-8 expression was observed when comparing standard and high doses of HMO (*p* > 0.05). Magpix analysis of inflammatory cytokines indicated that 2′-FL, individually and in combination with 6′-SL, reduced the expression of pro-inflammatory TNFα when compared to the glucose control (*p* < 0.0001) across all doses. Similar findings were observed for expression of IL-8, IL-1β, IL-6 and IFNγ where treatment with 2′-FL, 6′-SL, and combinations thereof resulted in reduced levels of inflammatory cytokines compared to treatment with glucose (*p* < 0.05). These HMO treatments (2′-FL, 6′-SL, and 2′-FL + 6′-SL) resulted in similar inflammatory phenotypes to breastmilk-derived HMO (BM-HMO).

#### 3.3.3. Assessing the Optimal Ratio of HMO for Amelioration of Inflammation

To further understand the potential synergistic action between 2′-FL and 6′-SL in reducing intestinal inflammatory markers, these two HMOs were applied to the inflammatory model at various concentrations (0.2 g/L to 1.2 g/L) and various ratios (from 2:1 to 6:1) (Table 3). Levels of inflammatory cytokines, as measured by multiplex ELISA, are summarized in Table 4. Treatment with BM-HMO reduced expression of all cytokines measured (IL-1β, IL-6, IL-8, and TNF-α) when compared to treatment with DMEM supplemented with glucose (*p* < 0.05). Although the ratio of 2′-FL to 6′-SL can fluctuate based on the mother’s lactation stage and individual variation [42], we found that application of these HMOs at ratios similar to those in colostrum and transitional milk (between 2:1 and 4:1) was superior to treatment with a 6:1 ratio of 2′-FL: 6′-SL (representative of mature and late-stage milk). Interestingly, application of 0.6 g/L 2′-FL and 0.3 g/L 6′-SL (2:1 ratio, total = 0.9 g/L) significantly reduced expression of IL-1β and IL-8 when compared to the glucose control (*p* < 0.05), while application of 1.2 g/L 2′-FL and 0.2 g/L 6′-SL (6:1 ratio, total = 0.9 g/L) had no significant effect on the expression of these cytokines. Anti-inflammatory IL-10 was detected across all treatments of the 2′-FL and 6′-SL combination. The expression of IL-10 was not dependent on the ratio applied, with similar levels detected across all treatment groups (*p* > 0.05).

## 4. Discussion

In the realm of neonatal health, inflammatory disorders of the intestine present significant challenges, necessitating extensive research to understand their pathophysiology and develop prevention strategies [43]. Such disorders often arise from chronic inflammation, which can lead to intestinal mucosal barrier dysfunction, marked by diminished gut mucus thickness and heightened intestinal permeability [44]. This inflammation can be triggered by various perinatal insults, including bacterial colonization, excessive expression of toll-like receptor 4 (TLR4), and hypoxic stress [45]. It is widely recognized that human milk is the optimal nutrition source for the first six months of life, offering protection against various forms of neonatal intestinal inflammation [46]. Preterm infants fed human milk have a substantially lower risk of developing severe intestinal issues than those fed formula [47]. Research from in vitro tissue models, in vivo animal studies, and human mother–infant cohort studies highlights the protective role of HMOs, which are abundant in human milk but absent in formula [48]. Our research utilizes an in vitro co-culture model of intestinal epithelial Caco-2 cells and THP-1 macrophages to explore whether synthetically produced HMO blends can mimic the protective effects of breastmilk against inflammation associated with intestinal disorders.

Following the approach of Kampfer et al., we used *E. coli*-derived LPS as an inflammatory stimulus, directly applied to THP-1 cells to elicit an inflammation-like response [41]. Caco-2 cells express the LPS-binding receptor CD14 and Toll-like Receptor (TLR)-2 but lack TLR4, essential for LPS-induced signalling [49]. This co-culture model mimics bacterial antigens crossing the intestinal epithelial cell barrier, activating gut-associated lymphoid tissue cells. Priming THP-1 cells with LPS and IFN-γ induced an inflammation-like response, replicating key mechanisms of inflammatory disorders. This Caco-2 inflammation model exhibits hallmarks of inflammatory disorders of the intestine, including a disrupted epithelial barrier, high pro-inflammatory cytokine levels, and cell death [50].

Bacterial invasion and the presence of metabolites such as LPS in the intestinal mucosa stimulate the production of inflammatory cytokines, including IL-8, IL-1β, IL-6, IFN-γ, and TNFα. These cytokines further enhance intestinal permeability, perpetuating a cycle of inflammation [51,52]. In intestinal inflammation scenarios, IL-8 attracts neutrophils, releasing enzymes and reactive oxygen species that damage tissue. IL-1β and TNFα increase intestinal permeability, promoting inflammation and cellular apoptosis. Elevated IL-6 levels facilitate T and B cell differentiation and the production of acute-phase proteins, intensifying inflammation. IFN-γ exacerbates inflammation by activating macrophages. IL-10, however, modulates the immune response, balancing defense and tolerance, thereby suppressing excessive inflammation and protecting against tissue damage [53,54,55].

This current study was designed to target these pro-inflammatory cytokines to investigate the direct, non-prebiotic effect of blends of 2-HMO, 3-HMO, 4-HMO, and 5-HMO on intestinal epithelial cell responses associated with inflammatory disorders and compared to the anti-inflammatory effects of breastmilk-derived HMO. As expected, the accumulation of LPS-IFNy-induced pro-inflammatory cytokines was reduced most significantly by breastmilk-derived HMO. HMOs isolated directly from breastmilk may provide superior protection against inflammation compared to a synthetic blend of HMOs because of the greater structural diversity, synergistic effects, and presence of minor HMOs which may provide a more comprehensive anti-inflammatory response than a limited synthetic HMO blend [56,57]. The breastmilk-derived HMO included in this study may contain up to 200 distinct HMO structures, each with unique functions and interactions. This diversity of oligosaccharides allows for a broader range of biological activities. Synthetic blends, typically limited to a few HMOs, cannot replicate this complexity, potentially reducing the protective effect [56,57]. It has been assumed that blending multiple HMOs can provide a broader range of potential benefits by targeting different pathways involved in inflammation. Remarkably, we show that increasing the range and diversity of synthetic HMO did not improve inflammatory signatures in this Caco-2 model. Among the synthetic blends, the highest anti-inflammatory effects were observed in a combination of two HMOs: 2′-FL and 6′-SL. These findings align with previous studies which indicated that specific oligosaccharides such as 2′-FL and 6′-SL, when applied individually, can modulate multiple functions of intestinal epithelial cells, in vitro, in vivo and ex vivo [36,58,59,60,61,62,63,64]. However, in comparison to these studies, we demonstrate that ratio- and concentration-specific combinations of these two HMOs, which represent the most abundant fucosylated and sialylated HMO structures, can offer even greater anti-inflammatory effects by targeting the suppression of a greater multitude of cytokines. All tested concentrations of 2′-FL and 6′-SL in combination reduced the expression of IL-1β and TNF-α, while only the higher tested concentrations of 2′-FL (1.2 and 2.4 g/L) and 6′-SL (0.4 and 0.8 g/L) inhibited LPS-induced IL-8 release, well within the range of 2′-FL and 6′-SL levels found in human milk (2′-FL: 1.65 g/L in mature milk to 3.18 g/L in early-term milk, 6′-SL: 0.3 g/L in mature milk to 0.71 in early-term milk) and can thus be considered physiologically relevant. Although the ratio of 2′-FL to 6′-SL can fluctuate based on the mother’s lactation stage and individual variation [42], we found that application of these HMOs at ratios similar to those in colostrum and transitional milk (between 2:1 and 4:1) was superior to treatment with a 6:1 ratio of 2′-FL: 6′-SL (representative of mature and late-stage milk) [42]. In contrast to prior research, our findings indicate that LNnT did not contribute to a reduction in inflammation. Previous studies have reported that LNnT can suppress Th1-type responses, which produce cytokines like IFN-γ, IL-2, and TNF-β, all of which play roles in immune and inflammatory reactions [65]. Additionally, research by Farhadihosseinabadi et al. demonstrated that LNnT promoted a type 2 anti-inflammatory response in a mouse model, where the treated group exhibited higher levels of IL-10, IL-4, and IL-13 compared to the control group (administered PBS) [66]. The discrepancies observed between this study and previous studies, demonstrating anti-inflammatory effects, is likely due to the different models applied.

We propose that the increased anti-inflammatory effects observed with the combination of 2′-FL and 6′-SL might be due to their capacity to interact with epithelial cells through distinct but complementary pathways, thus modulating the immune response. This synergistic blend could exert anti-inflammatory effects in the Caco-2 and THP-1 co-culture model through several mechanisms and receptors associated with immune modulation, primarily involving interactions with pattern recognition receptors (PRRs) such as toll-like receptors (TLRs) and nucleotide-binding oligomerization domain-containing protein 2 (NOD2), engagement with immune-associated lectins, and reinforcement of barrier integrity. This synergy may improve gut health, enhance immune resilience, and reduce inflammation more effectively than either HMO alone.

The first evidence of intact HMOs in the urine of breastfed infants was reported by Rudloff and colleagues in 1996 [67], and subsequent studies have shown absorption rates of 1–5% of HMOs from the gastrointestinal tract into the bloodstream [68,69,70]. HMOs are known to influence immune cell interactions, promoting balanced inflammatory responses [32]. Human lectins (glycan-binding proteins) are particularly relevant, as they directly interact with HMOs and other milk glycans. To explore these interactions, Noll et al. screened the glycan-binding potential of C-type lectins and sialic acid-binding immunoglobulin-like lectins (Siglecs) with over 200 human milk glycans [71]. Their findings showed that 2′-FL and 3-FL bind to dendritic cell-specific intercellular adhesion molecule-3-grabbing non-integrin (DC-SIGN), while LNT did not [71]. On the other hand, sialyllactoses (including 6′-SL and 3′-SL) have been shown to bind to sialoadhesin (Siglec-1) and also to Siglec-3, Siglec-5, Siglec-7, Siglec-9, and Siglec-10 [72,73,74,75]. Studies have also demonstrated that galectins bind selectively to siallylactoses, although with a significantly higher affinity for 3′-SL when compared with 6′-SL [76,77].

In addition, the anti-inflammatory effect of the 2′-FL and 6′-SL combination used here could be mediated through interactions with toll-like receptors (TLRs). TLRs act as primary sensors of microbial products and activate signalling pathways through NF-κB, resulting in the modulation of cytokine release [78,79]. Several classes of TLRs with HMO binding specificity have been described in the literature. For example, a study using THP-1 macrophages demonstrated that 2′-FL and 6′-SL (as well as 3-FL and LNnT) suppressed TLR-5 and TLR-7 activation [80]. Asakuma et al. (2010) showed that 3′-SL, 6′-SL, and 6′-galactosyllactose (6′-GL) influenced both TLR-2 and TLR-4 expression [81]. Anti-inflammatory effects via interactions with TLR4 have also been demonstrated for 2′-FL [34]. This is relevant because Gram-negative bacteria initiate mucosal inflammation by binding LPS to TLR-4 on intestinal epithelial cells (IECs) [82]. In silico modeling has predicted that 2′-FL and 6′-SL can dock onto TLR-4, thereby inhibiting it’s signaling [59]. In mouse and piglet models of NEC, 2′-FL and 6′-SL have been shown to reduce inflammation and NEC symptoms, partly by inhibiting TLR-4 signaling, which is implicated in NEC onset [59]. In contrast, pooled goat milk oligosaccharides, inulin, and synthetic fructo- and galacto-oligosaccharides were shown to stimulate immune responses and induce chemokine release through TLR-4 and NFκB activation. Additionally, 2′-FL has been found to inhibit *E. coli*-induced IL-8 release by downregulating the LPS receptor complex component CD14 [34], a mechanism which has also been validated in vivo. As CD14 serves as a co-receptor for LPS and is involved in TLR-4 signaling, this may lead to decreased intestinal inflammation following LPS exposure.

Mechanistically, it is also plausible to hypothesize that the observed immunomodulatory effects imparted by this combination of 2′-FL and 6′-SL may be due to these HMOs supporting the maturation of the intestine by increasing barrier integrity and stimulating mucin expression. Although changes in TEER were not significant, the barrier-enhancing effects were more pronounced for this HMO combination, especially at higher concentrations. The intestinal epithelium is crucial for gut homeostasis, as its cells and the overlaying mucus layer form a barrier separating the lumen from the submucosa and systemic circulation [83]. An impaired intestinal barrier allows luminal contents, including bacteria, to translocate into submucosal tissues, causing inflammation and potentially leading to gastrointestinal and systemic diseases [84]. Barrier impairment is especially concerning in preterm infants as it increases NEC risk. Breastfed infants show a quicker reduction in intestinal permeability compared to formula-fed infants, suggesting that components in breastmilk aid intestinal maturation [85,86]. In vitro studies have demonstrated a capacity for 6′-SL, as well as 2′-FL, to support the development of a mature intestine [58,87]. In an animal study, breastmilk-derived HMO fractions increased MUC2 expression and reduced intestinal permeability in a neonatal mouse model of NEC [88]. HMOs were found to promote mucin expression by acting on chaperone proteins such as protein disulfide isomerase [88]. The results of this animal study are in line with findings from an in vitro study [89]. Here, the effects of HMOs on expression of goblet cell secretory genes in a colorectal cell model were assessed [89]. 2′-FL increased MUC2 expression under IL-13 exposure, while 3-FL upregulated both MUC2 and TFF3 expression under TNFα and IL-13 exposure [89]. Additionally, sialyllactose has been shown to promote the differentiation and growth of human intestinal epithelial cells, as evidenced by the upregulation of alkaline phosphatase expression [90]. Alkaline phosphatase plays a role in gut barrier maintenance, potentially by deactivating LPS through phosphate removal [91].

Overall, these studies confirm that 2′-FL and 6′-SL have a variety of direct effects on epithelial cell functions, particularly concerning bacterial infection and intestinal inflammation. These diverse effects are likely dependent on improvements in barrier function, inhibition of TLR-4, downregulation of CD14, and decreased activity of NFκB pathways. Further studies assessing the regulation and activation of genes along these immune-associated pathways are required to fully elucidate the mechanisms through which the beneficial effects of HMO combinations are elicited.

## 5. Conclusions

This study presents a co-culture model of Caco-2 intestinal epithelial cells and THP-1 macrophages to investigate the anti-inflammatory effects of human milk oligosaccharides (HMOs) against inflammatory disorders of the intestine. Our findings reveal that specific combinations of synthetic HMOs, particularly 2′-fucosyllactose (2′-FL) and 6′-siallylactose (6′-SL), effectively reduce pro-inflammatory cytokine levels. These results underscore the potential of targeted HMO blends in infant nutrition to mimic breastmilk benefits and prevent excessive inflammation. This study reinforces the concept that structurally different oligosaccharides have distinct biological activities and identifies, for the first time, that certain HMOs, i.e., 6′-SL and 2′-FL, modulate human epithelial cell responses related to inflammatory pathologies. Taken together, our results indicate that HMOs including 2′-FL and 6′-SL could be applied as functional food ingredients for maintaining intestinal function and preventing excessive inflammation. It is important to note that the specific interactions and outcomes can depend on the individual HMO structures, concentrations, and the experimental conditions. Ongoing research is continually refining our understanding of how different HMOs work individually and in combination to influence inflammation and other aspects of immune function.

## 6. Patents

WO2023203501—2′-FUCOSYLLACTOSE AND 6′-SIALYLLACTOSE FOR MODULATING THE IMMUNE SYSTEM

## Figures and Tables

**Table 1:** Concentrations of HMO applied during structure dependency assay.

Sample	HMO Concentration (g/L)					
	2′-FL	LNT	LNnT	6′-SL	LNFP I	Total
Glucose						3.8 *
BM-HMO						3.8 **
HMO5	1.2	0.8	0.6	0.4	0.8	3.8
HMO4	1.2	0.8	0.6		0.8	3.4
HMO3	1.2		0.6	0.4		2.2
2′-FL + LNFP I	1.2				0.8	2
2′-FL + 6′-SL	1.2			0.4		1.6
2′-FL	1.2					1.2
LNT		0.8				0.8
LNnT			0.6			0.6
6′-SL				0.4		0.4

* refers to concentration of glucose present in glucose-supplemented DMEM control. ** refers to concentration of breastmilk-derived HMO in BM-HMO-supplemented DMEM control. BM-HMO = breastmilk-derived HMO, 2′-FL = 2′-fucosyllactose, LNFP I = lacto-*N*-fucopentaose I, 6′-SL = 6′-siallyactose, LNT = lacto-*N*-tetraose, LNnT = lacto-*N*-neotetraose. The shading intensity corresponds to concentration levels, with darker green indicating higher concentrations of HMO.

**Table 2:** Concentrations of HMO applied during concentration dependency assay.

	Dosage	Relation to Regulatory	HMO Concentration (g/L)					
			2′-FL	LNT	LNnT	6′-SL	LNFP I	Total
Glucose	Low	NA						1.9 *
	Standard	NA						3.8 *
	High	NA						7.6 *
BM-HMO	Low	NA						1.9 **
	Standard	NA						3.8 **
	High	NA						7.6 **
HMO5	Low	0.5×	0.6	0.4	0.3	0.2	0.4	1.9
	Standard	1×	1.2	0.8	0.6	0.4	0.8	3.8
	High	2×	2.4	1.6	1.2	0.8	1.6	7.6
2′-FL and 6′-SL	Low	0.5×	0.6			0.2		0.8
	Standard	1×	1.2			0.4		1.6
	High	2×	2.4			0.8		3.2
2′-FL	Low	0.5×	0.6					0.6
	Standard	1×	1.2					1.2
	High	2×	2.4					2.4
6′-SL	Low	0.5×				0.2		0.2
	Standard	1×				0.4		0.4
	High	2×				0.8		0.8

* refers to concentration of glucose present in glucose-supplemented DMEM control. ** refers to concentration of breastmilk-derived HMO in BM-HMO-supplemented DMEM control. 2′-FL (2′-fucosyllactose) and 6′-SL (6′-siallylactose) were applied at low, standard, and high doses equivalent to 0.5×, 1×, and 2× the maximum regulated dose (according to the European Union List of authorized novel foods). The shading intensity corresponds to concentration levels, with darker green indicating higher concentrations of HMO.

**Table 3:** Concentrations of HMO applied during ratio dependency assay.

Treatment	HMO Concentration (mg/mL)			Ratio
	2′-FL	6′-SL	Final	2′-FL: 6′-SL
Glucose			1.6 *	
BM-HMO			1.6 **	
2′-FL, 6′-SL combination	1.2	0.4	1.6	3:1
	1.2	0.3	1.5	4:1
	1.2	0.2	1.4	6:1
	0.6	0.3	0.9	2:1
	0.6	0.2	0.8	3:1
2′-FL	1.2		1.2	
	0.6		0.6	
6′-SL		0.4	0.4	
		0.3	0.3	
		0.2	0.2	

* refers to concentration of glucose present in glucose-supplemented DMEM control. ** refers to concentration of breastmilk-derived HMO in BM-HMO-supplemented DMEM control. 2′-FL = 2′-fucosyllactose, 6′-SL = 6′-siallylactose. The shading intensity corresponds to concentration levels, with darker green indicating higher concentrations of HMO.

**Table 4:** Release of pro-inflammatory cytokines (TNFα, IL-1β, IL-6, IL-8) and TEER (ohm·cm^2^) following 24 h treatment of HMO.

Treatment	Concentration (mg/mL)			Ratio	Cytokine Concentration (pg/mL)															TEER (ohm·cm^2^)		
	2′-FL	6′-SL	Total	2′-FL: 6′-SL	TNFa			IL-1β			IL-6			IL-8			IL-10					
BM-HMO			1.6		2295	±	289.7 ^c^	338.2	±	33.78 ^de^	350.3	±	44.75 ^c^	7207.0	±	1738.22 ^d^	285.6	±	36.35 ^ac^	1002.7	±	151.48 ^a^
Glucose			1.6		4165	±	716.3 ^a^	528.9	±	76.28 ^a^	515.4	±	37.65 ^a^	17,907.6	±	4091.36 ^ab^	305.7	±	73.54 ^ab^	781.7	±	95.09 ^b^
2′-FL, 6′-SLcombination	1.2	0.4	1.6	3:1	2423	±	366.4 ^c^	323.5	±	60.07 ^e^	425.7	±	66.35 ^ac^	7197.0	±	1198.30 ^d^	353.3	±	94.15 ^a^	957.7	±	125.59 ^ab^
	1.2	0.3	1.5	4:1	2397	±	237.6 ^bc^	336.7	±	29.48 ^ce^	489.4	±	57.13 ^ac^	8874.7	±	3159.06 ^cd^	350.3	±	29.87 ^a^	965.2	±	117.83 ^ab^
	1.2	0.2	1.4	6:1	2401	±	601.89 ^bc^	412.2	±	35.81 ^ae^	492.1	±	43.63 ^ac^	10,353.0	±	3150.69 ^bd^	350.8	±	69.29 ^ac^	855.5	±	131.16 ^ab^
	0.6	0.3	0.9	2:1	2769	±	564.30 ^bc^	321.1	±	88.95 ^de^	491.1	±	92.87 ^ab^	8404.4	±	1826.54 ^cd^	287.5	±	58.64 ^ac^	920.5	±	89.39 ^ab^
	0.6	0.2	0.8	3:1	2647	±	618.41 ^bc^	378.8	±	68.18 ^be^	552.1	±	21.88 ^a^	13,439.3	±	4449.65 ^ad^	277.6	±	72.77 ^ac^	880.5	±	70.40 ^ab^
2′-FL	1.2		1.2		2389	±	350.03 ^c^	376	±	88.39 ^abc^	464.7	±	113.93 ^ab^	10,791.7	±	3017.57 ^bd^	95.1	±	18.65 ^c^	955.7	±	94.31 ^ab^
	0.6		0.6		2979	±	927.92 ^bc^	487	±	91.04 ^be^	481.3	±	47.50 ^ab^	14,515.3	±	3097.11 ^abc^	80.2	±	9.25 ^c^	886.5	±	64.35 ^ab^
6′-SL		0.4	0.4		2741	±	464.5 ^bc^	445.3	±	55.64 ^abcd^	384.1	±	39.64 ^a^	10,542.2	±	2908.20 ^bd^	273.6	±	64.07 ^ac^	911.5	±	101.89 ^ab^
		0.3	0.3		2460	±	501.64 ^c^	503.5	±	138.80 ^ab^	496.7	±	26.59 ^b^	16,151.1	±	3327.28 ^abc^	211.6	±	41.32 ^bc^	871.2	±	84.20 ^ab^
		0.2	0.2		3564	±	993.39 ^ab^	512.6	±	108.16 ^ae^	461.1	±	53.15 ^ab^	18,472.2	±	8935.83 ^a^	197.6	±	69.78 ^bc^	756.2	±	77.08 ^ab^

Values within a column with common letter = *p* > 0.05, calculated using Tukey’s multiple comparisons test. Analysis was performed using technical triplicate data from biological triplicate experiments.

## Data Availability

The authors confirm that the data supporting the findings of this study are available within the article and/or its Supplementary Materials.

## Supplementary Materials

The following supporting information can be downloaded at: https://www.mdpi.com/article/10.3390/biom14121481/s1, Figure S1: Pie chart showing the relative abundance of the major oligosaccharides in breastmilk-derived HMO.
