# Supplementary material for: Tailored Combinations of Human Milk Oligosaccharides Modulate the Immune Response in an In Vitro Model of Intestinal Inflammation
Source: Biomolecules. 2024 Nov 21;14(12):1481. doi: 10.3390/biom14121481 (PMC11727556; doi:10.3390/biom14121481)
Supplement: Supplementary file 1 [file biomolecules-14-01481-s001.zip › biomolecules-3220417-supplementary.pdf]

Supplementary Figure S1

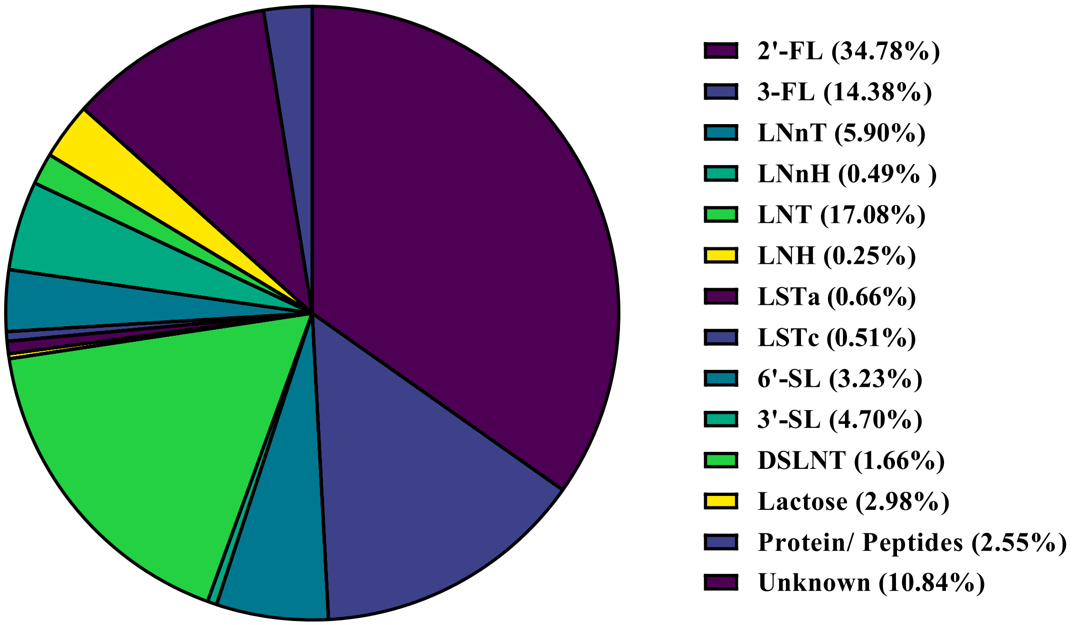

Figure S1: Pie chart showing the relative abundance of the major oligosaccharides in breastmilk-derived HMO which were detected via HPAEC-PAD analysis by comparing to the following external HMO standards: lactose, 3-fucosyllactose (3-FL), 2’-fucosyllactose (2'-FL), lacto-N-neotetraose (LNnT), lacto-N-neohexaose (LNnH), lacto-N-tetraose (LNT), lacto-N-hexaose (LNH), sialyllacto-N-tetraose a (LSTa), sialyllacto-N-tetraose a (LSTc), 6’-siallylactose (6'-SL), 3’-siallylactose (3'-SL), disialyllacto-N-tetraose (DSLNT). Data represents average concentrations of technical duplicate data from biological triplicate experiments
